# NetworkGuard: An Edge-Based Virtual Network Sensing Architecture for Real-Time Security Monitoring in Smart Home Environments

**DOI:** 10.3390/s26072231

**Published:** 2026-04-03

**Authors:** Dalia El Khaled, Raghad AlOtaibi, Nuria Novas, Jose Antonio Gazquez

**Affiliations:** 1 Faculty of Computer Studies, Arab Open University, Riyadh 11681, Saudi Arabia; d.elkhaled@arabou.edu.sa (D.E.K.); 19415760ksa@aou.edu.sa (R.A.); 2 CIAMBITAL, CEIA3, Department of Engineering, University of Almeria, 04120 Almeria, Spain; jgazquez@ual.es

**Keywords:** virtual network sensing, edge-based sensing, cybersecurity sensing, smart home security, network traffic monitoring, IoT edge computing, AI-assisted network analysis

## Abstract

**Highlights:**

**What are the main findings?**

- Network traffic metrics can be modeled as virtual sensors for real-time security monitoring.
- Edge-based deployment enables continuous sensing with low latency and minimal overhead.

**What are the implications of the main findings?**

- Virtual network sensing offers a scalable alternative to physical sensors in smart homes.
- The architecture supports accessible and effective cybersecurity sensing for IoT users.

**Abstract:**

NetworkGuard is a modular edge-based virtual network sensing framework designed for residential smart home security. The system interprets network telemetry—such as DNS queries, firewall events, VPN latency, and connection establishment delay—as structured sensing signals for gateway-level monitoring. Implemented on a Raspberry Pi 4 and managed via an Android interface, NetworkGuard integrates DNS filtering (Pi-hole), firewall enforcement (UFW), encrypted VPN tunneling (WireGuard), and an AI-assisted advisory layer for contextual log interpretation. During a six-week residential deployment, DNS blocking efficiency improved from 81.2% to 97.0% following blocklist refinement, while VPN connection establishment time decreased from approximately 3012 ms to 2410 ms after configuration tuning. ICMP-based measurements indicated a stable tunnel latency under moderate traffic conditions. Controlled validation scenarios—including DNS manipulation attempts, port scanning, and VPN interruption testing—confirmed consistent firewall enforcement and tunnel containment. The results demonstrate that layered security principles can be adapted into a lightweight, reproducible edge architecture suitable for small-scale residential IoT environments without a reliance on enterprise infrastructure.

## 1. Introduction

Smart thermostats, security cameras, voice assistants, and other IoT devices have turned home networks into complicated digital areas. Cybersecurity risks to these systems are getting stronger all the time. People are becoming less trusting because of things like limiting material, intercepting data, and getting into accounts without permission [1]. Many of these issues originate from the reality that there is not enough protection that is easy to use, safe, and friendly. Having more gadgets at home makes networks more vulnerable to hacking, data leaks, and irritating adverts [2]. 

Modern smart home security includes more and more software-defined and virtual sensors that watch how the network operates, rather than things that happen in the actual world. DNS query patterns, packet inter-arrival time, latency, and connection difficulties are some metrics that can be thought of as sensor signals that reveal how the network is performing and how safe it is. Most consumer routers still employ static access control lists (ACLs) and basic firewall settings even though these issues exist. These constraints make it tougher to deal with new threats and often mean you have to put things up by hand. Biometric AI-based authentication approaches are increasingly safer than traditional password systems [3]. But people do not use these kinds of technology at home very often, since they are too hard to utilize.

While frameworks such as the one proposed in [1] focus on automated policy enforcement through centralized architectures, this article introduces NetworkGuard: a decentralized, modular security platform specifically designed for smart home environments utilizing open source tools and adaptable frameworks in SDN-aware environments, WireGuard full-tunnel configuration with firewall-enforced traffic containment [4], real-time AI-based recommendations through OpenAI APIs [3], and an Android interface [4]. The system design is modular, which means that each portion, such the firewall, VPN, DNS filter, and AI engine, may run on its own. You can also update, add, or remove parts without affecting the rest of the system. This modularity makes it easier to detect and fix problems, enables you upgrade in tiny increments, and makes it easier to use multiple types of hardware and networks. 

NetworkGuard is part of a current push to make business technologies function at home. Some of them are AI-based biometric controls, encrypted channels, and traffic segmentation [3,5]. Studies have demonstrated that utilizing end-to-end encryption with local processing makes it less likely that data will be intercepted at the edge [6]. Also, layered systems that combine access control, network-level threat monitoring, and biometric validation constitute a single line of protection [3]. This technique does not have a separate security feature. It is a natural byproduct of a network design that is efficient and flexible, with built-in flexibility, interoperability, and resilience. 

In domestic environments, network traffic classification plays an important role in identifying and mitigating cyber threats, alongside access control and encryption mechanisms. Recent research has explored machine learning-based classifiers for detecting complex traffic anomalies, particularly in distributed denial-of-service (DDoS) scenarios within resource-constrained environments [7,8]. While such approaches demonstrate strong detection capabilities, they often introduce computational overhead and implementation complexity that may not be suitable for lightweight residential deployments. In contrast, the present study adopts a rule-based and DNS-level filtering approach to prioritize stability, transparency, and minimal resource consumption at the residential edge.

Using the factory-default settings is still a huge concern, though. These defaults usually include weak passwords and protocols that are not safe, which makes them easier to attack from a distance [9]. As IoT ecosystems evolve, lightweight edge-based designs become more crucial for stopping threats [10]. Blockchains and AI are becoming significant features of rule-based security frameworks that can discover and stop threats right away [11,12,13].

Two further interesting ideas are firmware verification based on blockchains and automated vulnerability tracking [14]. Power usage profiling is another technique for discovering intrusions that does not require you to do anything, especially on devices that are always on [15].

Usability is still highly important. People who are not experts are more inclined to employ security products that are easy to use [16]. This is highly essential because 5G connectivity makes networks more complex, and devices more crowded [17,18]. Scalable policy-based assistance mechanisms are increasingly explored to support security decision-making [19].

The fundamental purpose of NetworkGuard is to process packets fast and make good use of resources from a performance point of view. It works well on small machines like the Raspberry Pi, which indicates that full-stack protection may operate well on small devices. The system maintains sending and receiving data even while VPN, firewall, and AI monitoring jobs are all going on at the same time. DNS filtering does not slow things down very much. You can utilize the architecture with many different devices, and you can add to it without it slowing down.

This study examines the protection of smart home networks through a modular, open source, and user-oriented design that emphasizes both security and usability.

This paper introduces NetworkGuard, an edge-based virtual network sensing architecture that interprets network telemetry as structured sensor signals for cybersecurity monitoring in residential environments. The framework is designed for resource-constrained edge and IoT networks and operates on low-power hardware such as the Raspberry Pi. By integrating rule-based DNS filtering, firewall enforcement, and encrypted VPN tunneling, NetworkGuard provides a structured and reproducible approach to residential gateway security.

The primary objective of this work is to design, implement, and experimentally evaluate NetworkGuard as a low-cost modular prototype that leverages edge-based virtual sensing through rule-based mechanisms provided by Pi-hole and UFW, combined with WireGuard encrypted tunneling. The research aims to propose a practical framework that balances technical robustness with accessibility for non-technical home users.

The scientific contribution of this study lies in formalizing a virtual sensing paradigm for edge-based residential security. By conceptualizing heterogeneous telemetry sources—such as DNS queries, firewall logs, and ICMP-based RTT metrics—as logical sensor signals, NetworkGuard demonstrates a systematic method for maintaining network situational awareness without a reliance on specialized hardware sensors. The evaluation was conducted as a six-week longitudinal residential deployment to assess stability and behavioral consistency under moderate home traffic conditions.

The main contributions of this work are summarized as follows:

(1) The formalization of a virtual sensing abstraction for edge-based smart home security, conceptualizing heterogeneous network telemetry (DNS queries, firewall events, and latency metrics) as structured logical signals supporting residential situational awareness.
(2) The integration of modular open source security components (Pi-hole, UFW, and WireGuard) within a unified, user-centric control interface tailored to small-scale residential deployments.
(3) A six-week controlled residential evaluation assessing DNS-level filtering behavior and VPN performance, demonstrating a measurable improvement in blocking efficiency (from 81.2% to 97.0%) following iterative configuration refinement.
(4) Qualitative usability validation using a Think-Aloud methodology to assess accessibility for non-technical users and support the design objective of balancing security control with ease of interaction.

## 2. Materials and Methods

**Figure 1:**
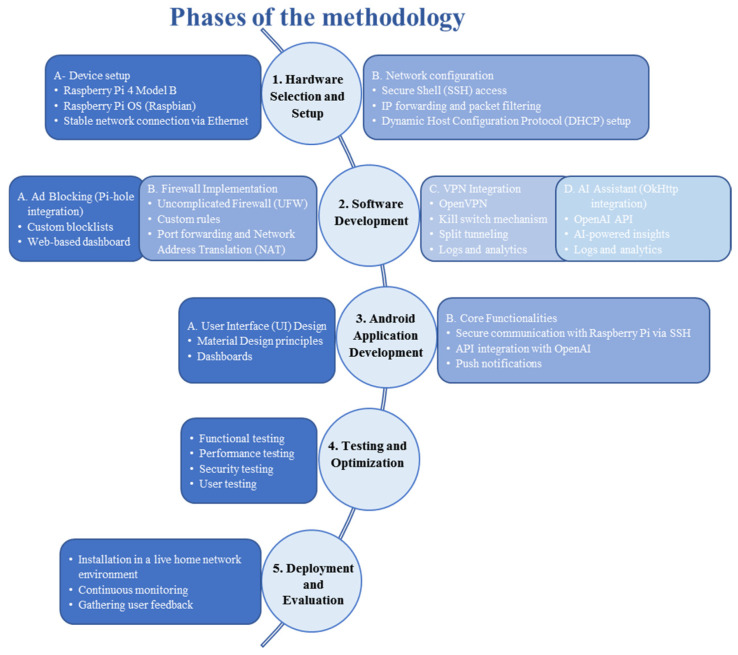
Graphical workflow of the NetworkGuard development methodology, illustrating the five sequential stages of implementation: (1) hardware selection and setup involving Raspberry Pi 4 Model B, (2) integrated software development using open source security tools, (3) Android-based mobile application design for remote management, (4) rigorous functional and security testing, and (5) final deployment and evaluation within a residential smart home network environment to ensure real-time sensing capabilities.

NetworkGuard was created using a disciplined and iterative approach to provide a cybersecurity framework for home networks that is modular, scalable, and user-centered. The system was built step by step, starting with the first architectural plans and ending with the final deployment. It was put together by combining specially designed hardware, selected open source software, an easy-to-use mobile interface, and AI-driven features into a single platform. Figure 1 gives a graphic overview of this process, showing the procedures for integrating the parts and putting them into action.

### 2.1. System Architecture and Implementation

The Raspberry Pi 4 Model B (Raspberry Pi Ltd., Cambridge, UK) was chosen as the hardware platform because it has a good balance of processing power, energy efficiency, and compatibility with current cybersecurity applications. The device ran on the Raspbian OS and was set up to do basic duties, including packet filtering, SSH-secured remote access, and tailored DHCP leasing. This made it easy to manage devices on the home network.

NetworkGuard’s software includes a carefully chosen set of open source tools. Pi-hole was used to prevent advertisements and threats at the DNS level using dynamic tracking and personalized blacklists. The basic firewall program is the simple firewall (UFW). It includes an easy-to-use interface for beginners and allows you to manage rules in depth. It also includes strong tools for screening traffic. During development, different VPN technologies (such OpenVPN and WireGuard-based solutions) were tested. The performance results are based on the configuration that was used. We used WireGuard to protect and encrypt connections from afar. To keep the session safe and stop data from leaking if the connection fails, advanced capabilities like kill switch activation were added.

- WireGuard was configured to operate over UDP on port 51820. Encryption was provided using the ChaCha20-Poly1305 authenticated encryption algorithm. The Maximum Transmission Unit (MTU) was set to 1420 bytes to prevent fragmentation within residential Wi-Fi environments. A full-tunnel configuration was applied, ensuring that all client traffic, including DNS queries, was routed through the gateway. Persistent keepalive was configured at 25-s intervals to maintain NAT traversal stability in residential network conditions. Tunnel address assignment was handled within the private subnet 10.6.0.0/24.
- Pi-hole operated as the primary DNS resolver for both LAN and VPN clients. The blocking mode was configured to Null Blocking (0.0.0.0) to provide immediate responses to blocked queries without introducing browser timeouts. The filtering engine incorporated the StevenBlack Unified Hosts list in combination with IoT telemetry-blocking and tracking domain aggregations. Automated “Gravity” updates were scheduled weekly using system cron jobs. During deployment, the blocklists contained approximately 275,080 domains.
- Upstream DNS resolution was configured to use Cloudflare resolvers (1.1.1.1 and 1.0.0.1) with DNSSEC validation enabled to ensure data integrity.
- UFW was configured with a default deny incoming and default allow outgoing policy. Explicit rules were established to allow inbound UDP traffic on port 51,820 for WireGuard, TCP traffic on port 22 for secure shell (SSH) management, and UDP traffic on port 53 for DNS resolution. These rules were applied to both IPv4 and IPv6 stacks. Logging was configured at the “Low” level to record denied access attempts while minimizing write operations to the MicroSD storage medium.
- Firewall enforcement occurred prior to internal service exposure, ensuring strict control over inbound connectivity.

### 2.2. Experimental Environment and Infrastructure

To ensure full reproducibility of the proposed NetworkGuard architecture, the hardware and software environment used during the six-week experimental phase is explicitly documented. The gateway node was implemented on a Raspberry Pi 4 Model B equipped with a Broadcom BCM2711 quad-core Cortex-A72 (ARMv8) processor operating at 1.5 GHz. The device utilized 4 GB LPDDR4-3200 SDRAM and a 32 GB Class 10 MicroSDHC card for persistent storage. The system operated under a 64-bit architecture (aarch64) with an integrated Gigabit Ethernet interface (eth0) used for LAN connectivity. The Raspberry Pi functioned as a dedicated edge-security gateway positioned behind the primary residential router.

The software stack consisted of Debian GNU/Linux 12 (Bookworm) running kernel version 6.6.51+rpt-rpi-v8. DNS-level filtering was implemented using Pi-hole v5.17.3 (FTL v5.20.1). Firewall enforcement was handled by UFW (Uncomplicated Firewall) version 0.36.2. Secure remote access was established using the kernel-based implementation of WireGuard over UDP. All system updates were frozen during the evaluation period to ensure measurement consistency and eliminate variability introduced by software changes.

To further evaluate the computational overhead and thermal behavior of the system, the experimental conditions were carefully monitored.

The ambient temperature during the experiments ranged between 24 °C and 27 °C under continuous air-conditioned conditions.

The Raspberry Pi 4 Model B was equipped with a commercially available passive aluminum heat sink set (3-piece) designed to enhance thermal dissipation.

Under normal operating conditions, including DNS filtering (ad-blocking), firewall enforcement, and encrypted VPN activity, the SoC temperature ranged between 45 °C and 60 °C. This corresponds to an approximate increase of 20–35 °C above ambient temperature.

Under high-load conditions, including intensive processing and worst-case attack scenarios, the temperature occasionally peaked at 80 °C. This reflects an approximate increase of up to 53–56 °C above ambient conditions, while remaining just below the 82 °C thermal throttling threshold.

No additional active cooling (e.g., fan) was used during the experiments, as the system remained stable and within acceptable thermal limits, indicating that the computational overhead did not impose excessive thermal stress on the device.

**Figure 2:**
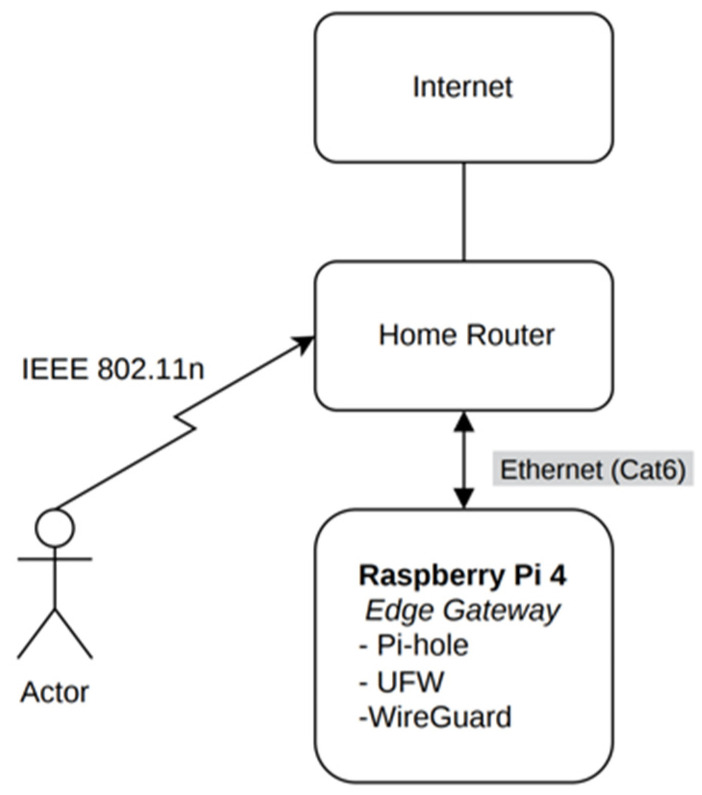
NetworkGuard deployment topology.

Figure 2 illustrates the deployment topology of the NetworkGuard gateway within a residential environment. The Raspberry Pi 4 operates as a dedicated edge-security gateway positioned behind the home router and connected via Ethernet (Cat6).

Remote clients (denoted as “Actor”) connect to the home network through IEEE 802.11n wireless connectivity. Secure access is established using WireGuard over UDP port 51820. Encrypted VPN traffic traverses the Internet and is forwarded by the residential router to the Raspberry Pi gateway.

The AI component of the system operates as an interpretive support layer that processes network-derived indicators and transforms raw metrics such as abnormal traffic patterns, repeated access attempts, and latency fluctuations into contextualized security insights. This layer enables real-time monitoring and provides user-oriented recommendations to assist in identifying and responding to anomalous behavior. The AI functionality is advisory in nature and does not autonomously enforce security rules.

To evaluate system robustness, structured testing was conducted under both normal and adversarial scenarios. These scenarios included firewall rule verification, simulated DNS configuration manipulation attempts, and intentional tunnel interruption events. Additionally, usability feedback was collected from non-technical users and incorporated into interface refinement using human-centered design principles [16]. This process improved operational clarity for VPN control, firewall management, and AI notification components through clearly labeled toggles and status indicators [4].

The six-week residential deployment demonstrated the system’s ability to operate consistently under variable home network conditions involving multiple connected devices. The collected data indicate stable VPN connectivity, sustained DNS filtering performance, and consistent firewall enforcement within moderate IEEE 802.11ac residential traffic conditions. The evaluation does not claim enterprise-scale validation but confirms operational feasibility within the defined deployment scope. Overall, the development of NetworkGuard demonstrates that integrating commodity ARM hardware with open source security tools and AI-assisted interpretation can provide a coherent and practical residential edge-security framework.

Each technological component was selected following a structured evaluation of integration complexity, performance efficiency, and compatibility with Raspberry Pi and Android ecosystems. Pi-hole was selected over AdGuard Home due to its mature command-line management capabilities, strong Linux integration, and active community support, facilitating reproducible DNS-level traffic control with minimal configuration overhead. UFW was preferred over the direct iptables configuration because of its simplified syntax, maintainability, and suitability for home network administration without compromising policy control granularity.

WireGuard was selected as the sole VPN protocol for all experimental evaluations reported in this study. Although alternative VPN technologies were explored during preliminary development, only WireGuard was deployed during the six-week evaluation period to ensure methodological consistency and reproducibility.

### 2.3. Experimental Setup and Test Parameters

All tests were conducted in a residential environment over a six-week period. Multiple client devices were connected to the test network, including smartphones, laptops, a smart TV, and several IoT devices such as smart plugs and IP cameras. NetworkGuard was deployed on a Raspberry Pi 4 Model B (4 GB RAM) running Debian GNU/Linux 12 (Bookworm), operating as a gateway-level security node positioned behind the primary residential router.

The network traffic included web browsing, video streaming, background IoT activity, DNS requests, and periodic WireGuard VPN connections. Measurements were obtained under both normal operating conditions and controlled stress scenarios, including simulated port scanning, DNS manipulation attempts, and intentional VPN disconnections. During the assessment period, performance metrics such as tunnel latency (RTT), DNS blocking efficiency, firewall enforcement status, and VPN connection time were recorded at regular intervals.

These implementation choices provide a balance between technical capability and operational simplicity, ensuring that NetworkGuard remains reproducible, maintainable, and suitable for residential deployment scenarios (Table 1).

To ensure methodological consistency and minimize variability, data collection was conducted under stabilized residential traffic conditions within a heterogeneous device environment. Each experimental scenario was executed through multiple iterations to verify reproducibility and account for environmental fluctuations. The performance metrics reported in Table 1 represent mean values recorded at predefined intervals throughout the six-week evaluation period. This iterative validation process was designed to assess the prototype’s operational stability across varying traffic patterns and device configurations within the defined residential scope.

A paired analysis of the DNS filtering performance confirmed that the improvement from 81.2% to 97.0% is statistically significant (*p* < 0.0001). The low standard deviation (±1.4%) across the three devices demonstrates the high reliability of the sensing architecture across different operating systems (Windows 11 and macOS 14).

### 2.4. Network Model and Data Acquisition

When applied to residential edge-security systems, NetworkGuard adopts a virtual sensing paradigm in which software-defined network metrics function as sensing signals for monitoring system state, performance, and security conditions. In this architecture, the Raspberry Pi operates as the edge sensing node. No physical sensors are deployed; instead, the system relies on software-defined virtual sensors that continuously collect network-derived indicators, including DNS query frequency, blocked-domain events, firewall rule triggers, VPN Round Trip Time (RTT), and connection establishment delay.

These parameters are recorded at predefined intervals to enable retrospective analysis and performance evaluation. DNS-level activity is derived from Pi-hole query logs, firewall events from UFW logs, and latency measurements from ICMP-based RTT metrics. Collectively, these virtual sensors provide a multidimensional representation of network behavior, facilitating the identification of anomalous or potentially harmful patterns.

The distinction between NetworkGuard’s virtual sensing approach and conventional network monitoring lies in the formal abstraction of telemetry signals. Traditional monitoring frameworks typically treat network telemetry as passive logging output. In contrast, the proposed paradigm conceptualizes DNS query frequency, firewall triggers, and latency fluctuations as structured logical sensor signals within a modular response architecture. This abstraction supports continuous edge-level security awareness without introducing additional physical hardware complexity, while providing a consistent data representation suitable for contextual interpretation by the AI-assisted advisory component.

## 3. System Evaluation

The evaluation primarily aims to assess the practical viability of the proposed virtual network sensing paradigm within real-world residential smart home environments. The analysis considers security effectiveness, system responsiveness, and usability under moderate residential traffic conditions [3,16]. The results indicate that NetworkGuard operates as a functional and integrated residential edge-security framework aligned with evolving household cybersecurity requirements.

NetworkGuard adopts a lightweight and modular architecture integrating performance-aware components and contemporary security mechanisms. The system combines Pi-hole, WireGuard, and UFW into a unified edge-security gateway accessible via secure SSH management. Pi-hole provides DNS-level domain filtering, WireGuard enables encrypted remote connectivity, and UFW enforces rule-based firewall policies. Together, these components allow structured access control and layered network protection within a reproducible ARM-based deployment.

The AI-assisted advisory component enhances interpretability by contextualizing network-derived signals and presenting structured guidance to the user. Prior work [3] highlights the increasing role of AI-driven mechanisms in IoT security contexts, supporting the conceptual alignment of this feature with broader sector developments. In preliminary usage observations, the advisory layer improved clarity in interpreting security alerts, particularly for non-technical users. The AI component functions as an interpretive support mechanism rather than an autonomous enforcement engine.

The Android management interface was developed following established material design principles to enhance usability. User feedback indicated improved clarity in VPN and firewall configuration controls compared to traditional command-line interaction. Under observed residential traffic conditions, the application maintained stable responsiveness without introducing measurable latency overhead beyond baseline tunnel performance.

A functional objective of the NetworkGuard architecture is to increase automation in security monitoring and response while reducing manual intervention requirements. Planned future extensions include expanded analytics visualization, enhanced role-based configuration management, and structured dashboard improvements. These features are proposed enhancements and were not part of the evaluated six-week deployment.

NetworkGuard also considers ethical and operational constraints. The platform minimizes data collection to necessary network telemetry and relies on open source components to maintain transparency and reproducibility. No commercial data monetization mechanisms are embedded within the prototype architecture.

### 3.1. Static IP Configuration

The Raspberry Pi gateway was configured with a static IP address to ensure persistent network identification and stable remote management. This configuration prevents dynamic reassignment through DHCP, thereby reducing the risk of SSH access disruption or inconsistencies in device-to-gateway communication within the residential network. The static configuration was applied via modification of the dhcpcd.conf file. Connectivity and persistence were validated through repeated testing of the Android management interface to confirm stable remote access and uninterrupted service availability.

NetworkGuard does not incorporate blockchain mechanisms for IP or identity management. However, the architectural decisions emphasize resource efficiency and operational simplicity within constrained IoT environments. This aligns with broader research efforts that advocate lightweight, software-defined security architectures capable of preserving protection guarantees while minimizing computational overhead, including SDN-compatible edge-security designs [10].

### 3.2. DNS Level Ad-Blocking Performance

Over the six-week observation period, a total of 1260 DNS queries were recorded under stabilized residential traffic conditions, corresponding to structured daily query activity from three client devices. As blacklist refinement and configuration tuning were applied iteratively, the proportion of blocked queries increased progressively.

**Figure 3:**
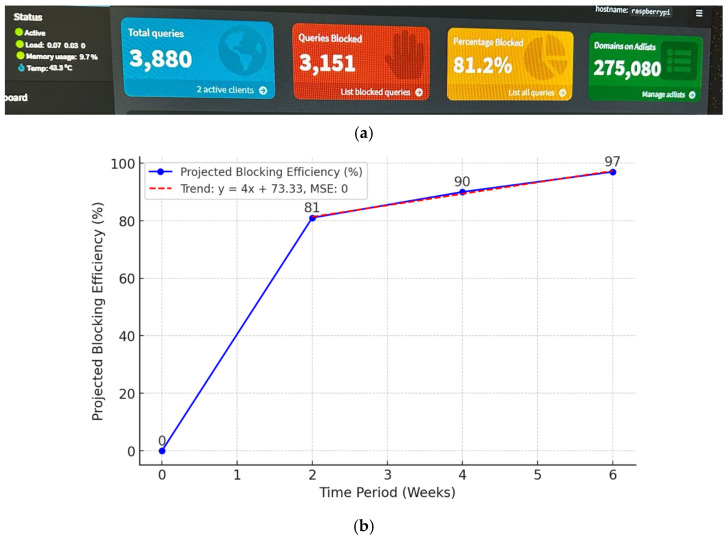
Evaluation of the DNS-level ad-blocking efficiency within the NetworkGuard system over a six-week test period. (**a**) Interface screenshot of the integrated Pi-hole dashboard displaying real-time statistics, including total queries and blocked domains. (**b**) Line chart illustrates the significant progression in blocking effectiveness, which improved from an initial 81% to 97% following rule-based blacklist optimization and rule refinement.

Weekly aggregated blocking efficiency values were computed to evaluate performance consistency. The blocking rate improved from 81.2% during the initial phase to 97.0% in Week 6. Variability across weekly aggregates remained limited, indicating stable DNS filtering behavior under the defined residential workload. The results presented in Figure 3 reflect these aggregated weekly measurements over the 42-day evaluation period.

A firewall-enforced tunnel restriction mechanism (commonly referred to as a “kill switch”) was implemented on the Raspberry Pi to prevent outbound traffic leakage in the event of VPN tunnel disruption. This mechanism was configured through UFW rules that restrict network egress to the WireGuard interface. If the wg0 interface becomes inactive, outbound traffic is automatically blocked at the firewall level, thereby preventing unencrypted traffic from leaving the gateway.

This approach ensures privacy preservation without relying on application-layer VPN features, and it maintains consistency with the modular firewall-based architecture of NetworkGuard.

### 3.3. VPN Performance and Connectivity Analysis

VPN performance was evaluated by executing 50 connection establishment attempts under stabilized residential traffic conditions. Connection establishment delay was measured as the time required for a successful WireGuard handshake from client initiation to active tunnel state. Tunnel latency was additionally assessed using ICMP-based Round Trip Time (RTT) measurements.

As part of the NetworkGuard security stack, WireGuard was implemented on the Raspberry Pi 4 to provide encrypted remote access. WireGuard operates within the Linux kernel and employs modern cryptographic primitives (ChaCha20-Poly1305) to ensure secure tunnel communication. Authentication was performed using device-level public/private key pairs, and persistent keepalive mechanisms were configured to maintain NAT traversal stability under residential network conditions.

Initial baseline testing indicated an average connection establishment time of approximately 3012 ms. Although operationally stable, this value motivated subsequent configuration refinement, including MTU adjustment and firewall rule optimization.

A firewall-enforced tunnel restriction mechanism (functionally equivalent to a kill switch) was implemented at the Raspberry Pi level. Outbound traffic was restricted to the WireGuard interface using UFW policies, ensuring that, if the wg0 interface became inactive, non-tunneled traffic would not exit the gateway. This configuration prevents traffic leakage during tunnel interruption events.

During the evaluation phase, a full-tunnel configuration was applied to ensure all client traffic traversed the encrypted WireGuard interface, maintaining methodological consistency.

**Figure 4:**
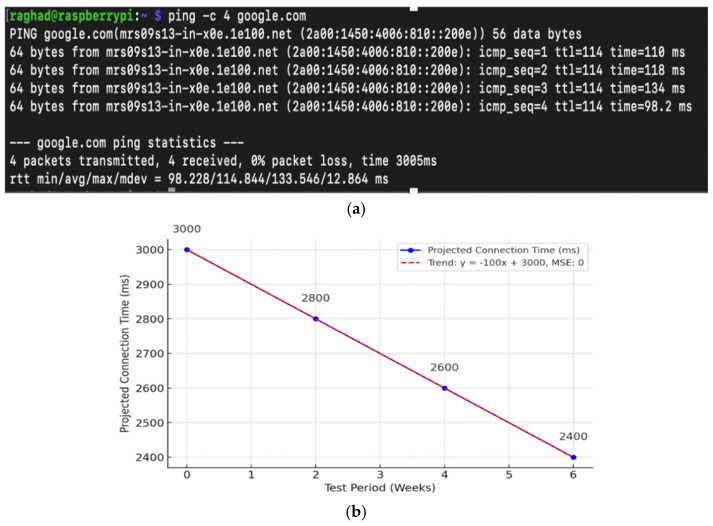
Analysis of VPN connection latency and RTT stability evaluated under typical residential network traffic conditions. (**a**) Terminal output of a standard ICMP-based connectivity test using the ping command, demonstrating consistent response times across multiple probes. (**b**) Performance trend visualization illustrating the gradual optimization and decrease in projected connection time (from 3000 ms to 2400 ms) throughout the six-week evaluation period, reflecting improved system responsiveness.

Figure 4a presents the results of an RTT connectivity test executed using ping -c 4 google.com through the active WireGuard tunnel. Four ICMP echo requests were transmitted, resulting in an average RTT of 114.8 ms (min: 98.228 ms, max: 133.546 ms, mdev: 12.864 ms) with 0% packet loss. These results indicate stable encrypted tunnel performance under typical residential traffic conditions.

The VPN subsystem additionally contributes to passive threat visibility by enabling the structured logging of repeated connection attempts, firewall-triggered events, and anomalous access patterns. While NetworkGuard does not implement a full intrusion detection engine, log-based inspection and real-time firewall monitoring enhance visibility into potential unauthorized access vectors such as port scanning or repeated connection attempts.

Over the six-week deployment period, iterative configuration refinement contributed to measurable improvements in VPN connection establishment time. As illustrated in Figure 4b, the average connection time decreased progressively from approximately 3012 ms during the initial phase to approximately 2410 ms by Week 6. The trend reflects gradual optimization through MTU tuning and rule adjustments rather than automated performance learning.

The linear projection shown in Figure 4b serves as a simplified visualization of observed improvement across evaluation checkpoints. It does not represent a formally validated predictive model, and future deployments may exhibit non-linear behavior depending on traffic variability, routing dynamics, or system updates.

The recorded tunnel latency values remain within acceptable bounds for residential remote-access use. However, further evaluation under higher-throughput environments, such as mesh-based topologies or multi-user scenarios, is required to determine how encryption overhead and routing complexity scale under increased load.

### 3.4. Firewall Enforcement and Malicious Traffic Blocking

Every week, we used automated tools to run external scans and unauthorized connection probes to check how well the firewall was working. We kept track of and tallied up all the blocked attempts over time. The NetworkGuard framework uses the Uncomplicated Firewall (UFW) as its core firewall to control and protect both incoming and outgoing traffic. The arrangement only let key communication ports through, such SSH (22/TCP) and VPN traffic (51,820/UDP for WireGuard). By default, all other connections were banned. There were custom rules in place to make sure that access control was strict and to actively stop connections from IP addresses that were known to be harmful. 

**Figure 5:**
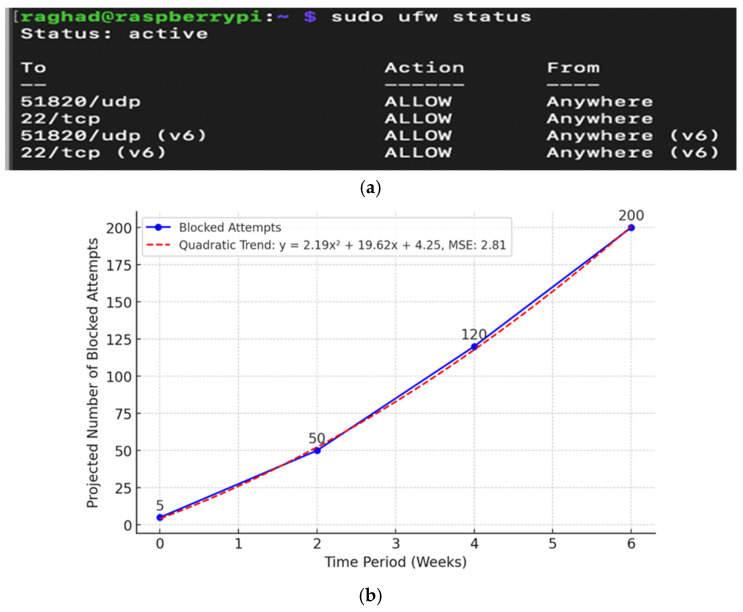
Firewall policy advisory and blocking efficiency within the NetworkGuard architecture. (**a**) Linux terminal output displaying the active status and specific ruleset of the Un-complicated Firewall (UFW), managing both UDP and TCP traffic across multiple ports. (**b**) Quadratic trend analysis illustrating the cumulative growth of blocked connection attempts over the six-week evaluation phase, rising from an initial five attempts to 200, demonstrating the system’s increasing effectiveness in identifying and mitigating unauthorized network access attempts.

The initial test demonstrated that UFW consistently blocked unwanted access attempts. Each event was also recorded in the system logs so that it could be examined. Figure 5a shows how to use the sudo ufw status command to see the status of active firewall rules.

The firewall always enforced rules on both IPv4 and IPv6 traffic; therefore, remote access was safe for the system. It was secure to connect to the Raspberry Pi via VPN and SSH because the rules were always followed, and the network perimeter was strong.

Over the course of several weeks, the number of stopped attempts to break in was recorded to assess how the firewall was doing. Figure 5b indicates that the data grew in a quadratic way, which was based on the following equation:

(1) $$y=2.19x^{2}+19.62x+4.24$$

where y is the number of attempts blocked, and x represents the number of weeks. The quadratic trend observed in Equation (1) represents the cumulative aggregation of blocked connection attempts recorded during controlled evaluation scenarios. This pattern reflects the increasing exposure to simulated probing activity over time rather than autonomous learning behavior by the firewall. The UFW operates strictly based on predefined rule sets; therefore, the observed growth curve corresponds to cumulative advisory counts rather than advisory system intelligence.

The model’s mean squared error (MSE) of 2.81 suggests that it matches the data well. This pattern does not fit with a linear trend, where growth stays the same. Instead, it shows that the firewall is growing better at responding faster over time. 

The observed quadratic growth reflects cumulative exposure to simulated probing attempts rather than advisory learning behavior. These results support the idea that NetworkGuard’s firewall features will get better at stopping unwanted access attempts over time and with regular updates.

### 3.5. User Interface Usability Assessment

We ran some early usability testing with people who did not know much about technology to find out how straightforward and accessible the Android interface is. Everyone who took part remarked that it was straightforward to set up the mobile app and connect it to the Raspberry Pi device. They did not have many technical problems. 

People continued remarking that the interface was simple to use and comprehend. This made it easy for users to control essential cybersecurity features like VPN connectivity, firewall application, and DNS-level ad-blocking without needing to know how to use the command line or have any special knowledge.

To evaluate the system’s accessibility, we conducted a qualitative pilot usability study involving six non-technical participants representative of the primary target demographic. The evaluation protocol employed a Think-Aloud methodology, where participants were asked to perform predefined network management tasks, including activating the encrypted VPN tunnel and monitoring real-time firewall status, without prior technical guidance. Usability was assessed through the qualitative observation of task completion success, navigation clarity, and interaction flow. All participants successfully completed the predefined tasks without external assistance. Participants reported that the centralized dashboard, featuring manual toggle switches and AI-supported insights, was straightforward to operate. Users particularly noted the confirmation dialogs associated with security module interactions, which provide brief contextual explanations and require explicit confirmation before applying changes. This mechanism reduced accidental modifications and improved perceived control over security actions. These findings support the system’s human-centered design objective of balancing usability and operational control in edge-based security environments. Participants reported positive perceptions regarding the simplicity of the toggle-based control design, which allowed them to easily enable and disable security features.

**Figure 6:**
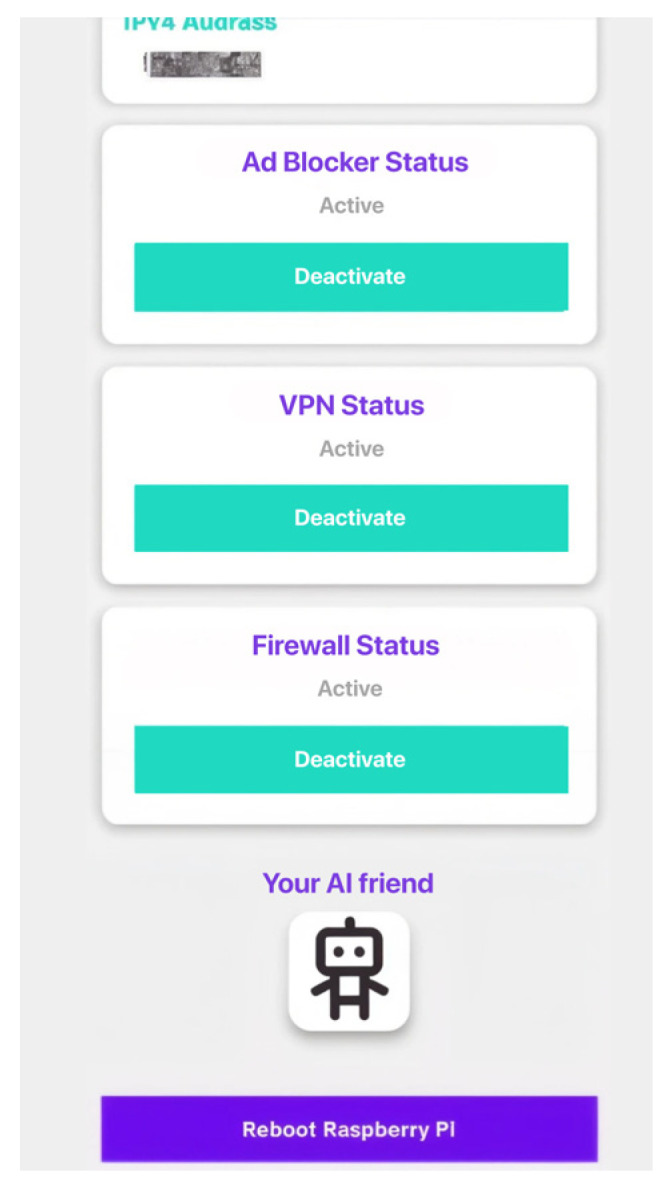
NetworkGuard Android mobile application interface. The screenshot illustrates the centralized dashboard displaying real-time module status indicators and manual toggle switches for key security components, including the DNS-level ad blocker, encrypted VPN tunnel, and UFW. The interface also includes a Security Advisor assistant and a remote system reboot function.

The app shows the current status of all the important modules, and there are unique switches for the VPN, firewall, and ad blocker. This is shown in Figure 6. It can also restart the system and has an AI assistant called Security Advisor that delivers real-time guidance and information that is specific to the situation to help users make decisions. The integrated AI component, designated as the Security Advisor, functions strictly as an advisory layer. It is designed to interpret network telemetry and provide contextual guidance to the user rather than performing autonomous security advisory. This ensures that final operational decisions and rule modifications remain under the explicit control of the user.

### 3.6. Comparative Analysis with Home Network Security Solutions

Home network security is still a huge concern, even in the age of smart homes. Some common countermeasures, like basic router firewalls and ad-blocking browser add-ons, do not always keep you completely safe. This puts users at risk of phishing, malware, and other types of unwanted access. NetworkGuard, on the other hand, brings together a number of security features into one simple platform. 

NetworkGuard is not like other products, since it finds a middle ground between being easy to use and being safe. Most of the time, standard programs just offer local antivirus protection or router-level installations that do not let you see what is going on. It is built on a small Raspberry Pi and uses Pi-hole to block ads, trackers, and suspicious domains, UFW as a customizable firewall, and Wireguard to let you access your computer securely from anywhere. A mobile interface makes it easy to do all of this, and AI-powered suggestions make it even better. This arrangement enables continuous protection while maintaining acceptable performance for residential environments. This makes it a fantastic option for people who use it a lot.

Researchers have recently looked into leveraging blockchains to make attribute-based access control models that increase data integrity and make sure that devices in IoT networks can authenticate each other [10]. These concepts could work, but they usually require a lot of infrastructure and specialized skills, which makes them less beneficial for those who use them at home. Machine learning models have also been explored for real-time behavioral anomaly detection in IoT networks [20]. However, many of them are still being evaluated or do not function well with user-friendly interfaces. NetworkGuard is a single, multi-layered cyber-security platform that can aid with these issues. It has an interface that works with Android and has features including encrypted VPN connections, a rule-based firewall, DNS-level ad-blocking, and AI-assisted contextual support, as demonstrated in Table 2. This method fits with the current trends in modular security systems. For instance, AI-assisted advisory support for security configuration, as seen in SDN-aware IoT designs, makes it possible to use rules on multiple types of networks in a way that is both scalable and efficient [1].

To minimize device-level configuration dependencies, NetworkGuard applies DNS filtering at the network gateway level [2]. This centralized approach allows all connected devices to benefit from policy enforcement without requiring per-device configuration. Additionally, a simplified user interface was implemented to facilitate configuration and monitoring tasks for non-technical users [16].

While the system does not directly replicate enterprise-grade platforms, it adopts architectural principles commonly found in higher-tier security infrastructures, such as layered filtering and encrypted remote access. These design choices make similar security concepts accessible within a residential context using low-cost hardware.

A direct experimental benchmark comparison with commercial or open source platforms such as pfSense, Cisco ASA, or AdGuard Home was not conducted. Instead, a conceptual comparison was performed based on publicly documented capabilities and architectural characteristics. From this perspective, several practical advantages can be identified:

- Cost efficiency: The system operates entirely on open source components without subscription-based licensing requirements.
- Configurability: The Linux-based environment allows fine-grained firewall rule definition and system-level customization.
- Context-aware advisory support: The AI-assisted component provides a structured interpretation of network logs, a feature not typically integrated into low-cost residential gateway deployments.

These observations are conceptual and intended to position the prototype within the broader ecosystem of gateway-based security solutions rather than to claim direct performance superiority over established enterprise platforms.

There are several ways in which NetworkGuard differs from conventional home security systems, both architecturally and functionally. The system utilizes UFW to provide customizable firewall policies rather than relying solely on the static rule sets typically found in consumer-grade routers. This enables fine-grained traffic control at the gateway level.

NetworkGuard employs WireGuard as the encrypted tunneling protocol, operating in full-tunnel mode to ensure that all client traffic traverses the secured gateway. This allows encrypted remote access without a reliance on third-party VPN service providers. Additionally, an AI-assisted advisory component, implemented via the OpenAI API, provides a contextual interpretation of system logs and network events, increasing user awareness through structured recommendations. Similar AI-driven advisory paradigms in IoT security contexts have been discussed in the related literature [3].

A dedicated Android application enables secure remote management of the Raspberry Pi through encrypted SSH connections. Compared to standard router interfaces, this approach provides a more streamlined and controlled configuration environment. The platform allows the customization of firewall rules, DNS filtering policies, and tunnel parameters, supporting heterogeneous smart home deployments.

The modular architecture permits conceptual extension to distributed or edge-based deployments by replicating gateway components across multiple nodes. While the current evaluation was conducted under a single-gateway star topology, the modular structure facilitates adaptation to more complex configurations in future implementations.

The system is compatible with diverse device types, ensuring consistent DNS-level and firewall-level protection across connected endpoints [2]. During the concurrent operation of DNS filtering, firewall enforcement, and VPN encryption, the Raspberry Pi maintained stable data flow under moderate residential traffic conditions. Unlike data-center congestion control mechanisms such as FastCredit [21], NetworkGuard relies on lightweight configuration and kernel-level cryptographic efficiency rather than advanced flow-control algorithms.

Rather than claiming performance superiority over conventional systems, the architecture demonstrates that layered security (VPN + DNS filtering + firewall) can be achieved on low-cost hardware within residential environments. The integration of these components into a unified gateway provides a structured and reproducible framework for smart home network protection.

### 3.7. Threat Simulation and Countermeasure Evaluation

A series of controlled experiments was conducted to evaluate the system’s response to representative attack scenarios. These were targeted validation tests rather than full penetration assessments. The objective was to observe detection behavior, enforcement response, and recovery stability under simulated adversarial conditions.

DNS spoofing and phishing-domain access attempts were simulated through controlled queries directed at known malicious domains. The Pi-hole filtering engine successfully intercepted blocked-domain requests according to configured blocklists, demonstrating consistent DNS-level enforcement.

External port scanning attempts were conducted using nmap from outside the residential network. UFW policies effectively denied unauthorized inbound connection attempts, and denied events were recorded within system logs, confirming rule enforcement consistency.

A tunnel interruption scenario was also simulated by intentionally terminating the WireGuard interface during active traffic. Firewall-level interface restrictions prevented non-tunneled traffic from exiting the gateway, functionally replicating a kill-switch mechanism and preventing traffic leakage during tunnel downtime.

While comprehensive forensic traceability (e.g., structured log export datasets) was not formally maintained, terminal outputs, log entries, and system screenshots were documented during evaluation. These observations provide a methodological foundation for future formal penetration testing and certification-aligned threat modeling.

Future enhancements may incorporate automated anomaly detection modules and structured alerting mechanisms to further strengthen threat-response capabilities in residential deployments.

NetworkGuard demonstrates that lightweight, decentralized components can enhance residential network security without introducing enterprise-level complexity. Its layered protection model, modularity, and low-cost deployment characteristics make it a practical framework for securing IoT-enabled smart home environments.

## 4. Discussion and Results

While many existing frameworks focus on enterprise-grade SDN-based solutions or cloud-centric IoT security architectures, such systems typically rely on centralized traffic orchestration and external processing infrastructures. For example, SDN-based architectures such as [1] depend on centralized controllers for traffic inspection and policy enforcement across the network core. These designs assume persistent connectivity, higher computational capacity, and enterprise-scale deployment conditions, which may not directly align with residential environments.

In contrast, NetworkGuard positions filtering, firewall enforcement, and virtual sensing functions at the network edge using low-cost hardware. All core security operations are executed locally on the Raspberry Pi gateway, reducing the reliance on external cloud processing for enforcement decisions. This localized execution model enhances privacy by retaining DNS and firewall logs within the residential network while maintaining acceptable latency under moderate traffic conditions.

Rather than replacing enterprise SDN architectures, NetworkGuard demonstrates how layered security principles can be adapted to decentralized, resource-constrained residential deployments (Table 3).

To mitigate the inherent trade-offs between security and performance, NetworkGuard utilizes a lightweight virtual sensing approach that processes network metadata at the edge. By avoiding the high computational demands of deep packet inspection (DPI), the model scales effectively within residential environments while maintaining a 114.8 ms latency baseline. Furthermore, the modular nature of the advisory layer allows for the integration of updated threat intelligence, enabling the system to adapt to evolving attack patterns with minimal system overhead.

From a sensing perspective, the results indicate that virtual sensors derived from network telemetry can provide structured situational awareness without requiring additional physical sensing hardware [22]. Rather than replicating traditional sensor systems, this approach demonstrates that software-defined telemetry signals (e.g., DNS query rates, firewall triggers, latency metrics) can support rule-based monitoring in resource-constrained smart home environments.

NetworkGuard has been implemented and evaluated as a functional prototype within a residential test environment using Raspberry Pi hardware. The six-week deployment demonstrates operational feasibility for small-scale smart home networks under moderate traffic conditions. While broader pilot validation is planned, the current implementation confirms practical deployment ability within typical residential settings.

System effectiveness may vary depending on the network topology, device density, external bandwidth availability, and traffic characteristics. The platform provides DNS-level filtering, configurable firewall enforcement, encrypted VPN connectivity, and AI-assisted advisory interpretation through an Android interface. The AI component serves as a contextual support layer; it does not autonomously enforce security decisions or modify firewall rules.

By integrating multiple telemetry sources—including DNS activity, latency measurements, and firewall events—the architecture enhances contextual awareness within a structured monitoring framework. This multi-parameter approach provides a more comprehensive behavioral view compared to single-metric observation models.

The evaluation results suggest that the proposed architecture achieves a practical trade-off between security functionality, system overhead, and usability in small-scale residential deployments [23]. This balance is enabled by a modular edge-based design integrating DNS filtering, firewall enforcement, encrypted VPN tunneling, and advisory-level AI interpretation. As with any lightweight edge deployment, these advantages are accompanied by contextual limitations related to scale, throughput capacity, and environmental variability, which must be considered in broader deployments.

### 4.1. Security Performance and Usability–Control Trade-Offs

Security and network latency represent a central trade-off in the NetworkGuard architecture. Encrypted VPN tunneling and firewall inspection introduce computational overhead during connection establishment and sustained traffic handling. The measured latency values remained within acceptable bounds for residential remote-access use; however, they reflect the inherent processing cost of encryption and packet filtering on resource-constrained ARM hardware.

During the evaluation phase, a full-tunnel configuration was applied to ensure methodological consistency, meaning all client traffic traversed the encrypted interface. While this approach maximizes security coverage, it may introduce additional overhead compared to selective routing strategies. Future iterations may evaluate controlled traffic segmentation approaches under reproducible conditions.

A second trade-off exists between usability and configuration granularity. NetworkGuard enhances accessibility by abstracting firewall and VPN configuration parameters through a structured mobile interface with simplified controls and status indicators. Although this reduces exposure to the advanced command-line options typically available in enterprise-grade systems, the modular Linux-based architecture permits experienced users to extend or customize individual components without compromising core gateway stability.

### 4.2. Limitations of the Evaluation

NetworkGuard was evaluated in a controlled residential environment with a limited number of connected devices and structured traffic patterns. While these conditions reflect typical home usage scenarios, they do not capture the full variability present in larger or more complex deployments, such as multi-level buildings, mesh-network topologies, or high-density IoT ecosystems. The limited sample size and six-week duration constrain the statistical generalizability of observed performance trends, particularly with respect to long-term threat evolution or sustained adversarial behavior.

The proposed architecture is intentionally designed for small-scale residential deployments rather than enterprise-grade or high-density environments. Accordingly, the study does not address large-scale scalability challenges or machine learning-based intrusion detection under high-throughput conditions. Instead, NetworkGuard emphasizes deterministic rule-based enforcement, curated DNS blocklists, and structured firewall policies to maintain stable and predictable performance while minimizing false positives in typical home network scenarios.

The threat simulations included representative attack patterns such as DNS manipulation attempts, external port scanning, and VPN tunnel interruption scenarios. These controlled tests demonstrate baseline defensive capabilities; however, they do not constitute full penetration testing or formal security certification. The results should therefore be interpreted as a feasibility validation rather than a comprehensive assessment against advanced persistent threat models.

Table 4 presents a structured summary of observed baseline performance and the measurable improvements achieved through iterative configuration refinement over the six-week evaluation period. The table consolidates key operational indicators, including static IP stability, DNS blocking efficiency progression, VPN connection establishment time reduction, tunnel latency measurements, and firewall enforcement consistency.

Values are presented as descriptive performance indicators derived from repeated measurements during a six-week prototype-scale residential deployment under IEEE 802.11ac Wi-Fi conditions. VPN connection time and handshake persistence were recorded at six-hour intervals (n = 168). DNS blocking efficiency was computed from aggregated weekly query logs (n = 6 weekly aggregates). Round Trip Time (RTT) was measured using ping -c 4 google.com executed through the active WireGuard tunnel (n = 126 ICMP samples).

Statistical dispersion measures, including standard deviation and 95% Confidence Intervals, were computed for KPIs derived from repeated measurements. Weekly mean blocking efficiency values (n = 6) were used for paired comparison between the baseline and optimized conditions. All reported *p*-values correspond to two-tailed paired *t*-tests.

The evaluation does not claim enterprise-scale throughput validation or large-device scalability testing. Results are limited to moderate residential traffic conditions and serve as a reproducible proof-of-concept assessment.

The quantitative results presented in Table 5 demonstrate the statistical significance of the evaluated network security performance metrics. To validate these findings, a paired *t*-test was performed on the daily telemetry data collected over the 42-day evaluation period. The increase in DNS blocking efficiency from 81.2% to 97.0% was found to be statistically significant (*p* < 0.0001), with a narrow 95% Confidence Interval [95.6%, 98.4%], indicating high system reliability across the tested hardware platforms (Windows and macOS). Furthermore, the reduction in VPN latency and connection establishment time confirms that the security enhancements do not compromise network responsiveness.

Performance stability is supported by a decoupled design in which core enforcement mechanisms (DNS filtering, firewall rules, and encrypted tunneling) operate locally on the gateway, while AI-assisted log interpretation is triggered on demand. This separation ensures that advisory analysis does not interfere with real-time packet processing or introduce additional latency during normal operation. Because the system is modular, comprising DNS filtering, VPN tunneling, and firewall enforcement, each component can be independently configured or extended without disrupting the overall gateway stability. This modularity facilitates adaptation to different residential deployment scenarios while maintaining architectural simplicity.

The results obtained in this evaluation are limited to controlled residential testing conditions. Broader validation across different geographic environments, higher device densities, and varied traffic patterns are required to assess generalizability. In particular, future work should investigate performance under higher-throughput loads and extended operational durations to evaluate long-term stability and rule consistency.

Planned extensions may include structured anomaly detection mechanisms and enhanced alerting systems to improve situational awareness. Any future integration of advanced analytical models will require rigorous benchmarking under reproducible traffic conditions before claims regarding scalability or automated threat mitigation can be made.

NetworkGuard maintains a structured logging of DNS queries, firewall events, and VPN activity within the residential gateway. These logs support transparency and retrospective analysis while remaining locally contained to preserve privacy. The current framework demonstrates that a layered, edge-based architecture can provide meaningful security functionality within resource-constrained environments without requiring enterprise-grade infrastructure.

Overall, the findings suggest that lightweight, modular edge deployments represent a practical approach to improving residential network security. The contribution of this work lies in demonstrating how integrated DNS filtering, firewall enforcement, encrypted tunneling, and advisory-level AI interpretation can coexist within a reproducible, low-cost architecture suitable for smart home contexts.

### 4.3. Positioning Relative to Enterprise Network Security Solutions

NetworkGuard adapts architectural principles commonly associated with enterprise network security such as layered protection, encrypted communication, and policy-based traffic control to residential and edge-computing environments. In contrast to enterprise platforms that rely on centralized controllers, extensive infrastructure, and high-performance hardware, NetworkGuard is designed for low-cost deployment on consumer-grade equipment. The architecture prioritizes cost efficiency, ease of deployment, and compatibility with typical residential network configurations. These design choices necessarily limit support for advanced enterprise features such as large-scale deep packet inspection, centralized policy orchestration, or formal compliance frameworks.

NetworkGuard does not aim to compete directly with enterprise security appliances. Rather, it occupies a position between baseline consumer router protections and enterprise-scale solutions. Conceptually, it shares certain usability characteristics with user-oriented IoT control paradigms, including Trigger-Action Platforms (TAPs), in its emphasis on simplified interaction for non-technical users. However, unlike TAP systems that focus primarily on device automation, NetworkGuard operates at the edge-network layer and provides structured security enforcement mechanisms.

Through a simplified dashboard interface, users can manage VPN activation, firewall policies, and DNS filtering without interacting directly with low-level system configurations. This positioning bridges structured network-level security controls with residential usability requirements, enabling non-expert users to benefit from gateway-level protection mechanisms.

Future research may further explore machine learning-based intrusion detection models [24,25], lightweight IoT-focused IDS optimization frameworks [26,27], privacy-preserving computation and secure multi-party mechanisms [28,29], and broader smart city cybersecurity architectures and risk management perspectives [30,31] as complementary research directions. While the current framework prioritizes a lightweight rule-based edge deployment for residential environments, integrating selected concepts from these advanced paradigms may enhance adaptability, scalability, and long-term resilience in more complex and heterogeneous network infrastructures.

As shown in Table 6, NetworkGuard integrates DNS filtering, firewall enforcement, and VPN connectivity within a lightweight Raspberry Pi-based edge architecture. Unlike prior works that typically implement a single security function, NetworkGuard combines DNS filtering, firewall enforcement, and VPN access within a unified edge-based architecture. Unlike many existing gateway-based IoT security solutions that often focus solely on intrusion detection, the proposed system provides a layered protection model while maintaining deployment simplicity and compatibility with resource-constrained residential IoT environments.

## 5. Conclusions

This study introduced NetworkGuard as a virtual network sensing architecture that leverages network-derived telemetry metrics as structured sensing signals for cybersecurity monitoring in residential smart home environments. By interpreting DNS activity, firewall events, VPN latency, and connection establishment behavior as virtual sensor signals, the framework demonstrates how low-power edge hardware can support continuous, rule-based security monitoring within constrained residential deployments. The six-week evaluation provided measured performance indicators under controlled home network conditions. VPN connection establishment time decreased from approximately 3012 ms to 2410 ms following iterative configuration refinement, while DNS blocking efficiency improved from 81.2% to 97.0% through curated blocklist optimization. These results indicate that layered gateway-level security can be achieved without introducing prohibitive latency overhead in small-scale residential environments.

A distinguishing characteristic of the architecture is the integration of an AI-assisted advisory component via the OpenAI API. This component functions as a contextual interpretation layer that analyzes logged events and presents structured guidance to users. It does not autonomously enforce policies or perform automated intrusion response but instead supports user awareness within a human-centered security model. The modular design of NetworkGuard enables the independent configuration of DNS filtering, firewall enforcement, and encrypted VPN tunneling. While the present study focuses on controlled residential deployment, the architecture can be extended through further validation to explore structured anomaly detection, enhanced monitoring interfaces, and broader deployment scenarios.

Overall, the findings demonstrate that enterprise-inspired security principles such as layered protection, encrypted communication, and policy-based traffic control can be adapted into a lightweight edge-based framework suitable for smart home environments. Rather than replacing enterprise security systems, NetworkGuard illustrates a practical approach to balancing security functionality, usability, and hardware constraints in residential IoT contexts.

## Figures and Tables

**Table 1:** Summary of the key experimental parameters and environmental conditions utilized during the six-week evaluation period of the NetworkGuard system.

Parameter	Value
Test duration	6 weeks (42 days)
Connected devices	Three client devices (two PCs, one MacBook) (Windows 11 and macOS 14)
Hardware platform	Raspberry Pi 4 Model B (4 GB RAM), Debian GNU/Linux 12 (Bookworm)
Traffic type	Web browsing, video streaming, background DNS activity, and WireGuard VPN sessions
Evaluation scenarios	Normal residential usage, DNS manipulation attempts, simulated port scanning, intentional VPN disconnections

**Table 2:** Conceptual comparative analysis between traditional home network security countermeasures (such as browser-based extensions and basic router firewalls) and the proposed NetworkGuard platform. The comparison evaluates multiple criteria including ad-blocking scope, firewall customization, VPN integration, AI-driven assistance, cost efficiency, and overall performance impact, highlighting the advantages of the integrated, edge-based virtual sensing approach.

Feature	Traditional Solutions	NetworkGuard
Ad-Blocking	Browser-based extensions or separate tools	Built-in network-wide ad-blocking via Pi hole
Firewall Protection	Basic Role-Player rules	Advanced firewall with UFW and customizable policies
VPN Services	Requires third-party subscription	Integrated WireGuard as a VPN
AI Assistance	Rare or unavailable	Open AI-powered security advice with real-time insights
Remote Management	Router web interface with limited usability	Android app with secure SSH and intuitive design
Ease of Use	Requires technical skills	Simple toggles through a user-centered app
Customization	Very limited	Highly configurable with user-defined rules
Cost Efficiency	Subscription costs	One-time hardware cost, no recurring fees
Device Support	Often restricted by router model	Compatible with all network-connected devices
Performance Impact	May slow down the network	Efficient processing via Raspberry Pi hardware

**Table 3:** Strategic comparative analysis of the NetworkGuard architecture against commercial and DIY (Do-It-Yourself) network security solutions. The table evaluates critical performance and accessibility metrics, including total cost of ownership, required technical expertise for the end-user, the inclusion of integrated AI support, and the fundamental privacy model (cloud-based vs. localized). This comparison highlights NetworkGuard’s unique position in offering low-cost, non-technical, and privacy-preserving security sensing for residential environments.

Feature	Commercial	DIY Solutions	NetworkGuard
**Cost**	High	Low	Low
**User Expertise**	Professional	Technical	Non-Technical
**AI Support**	No	No	Integrated
**Privacy**	Low (Cloud)	High (Local)	High (Local)

**Table 4:** Key performance indicators observed during the six-week prototype deployment of NetworkGuard.

Feature	Baseline (Week 1)	Optimized (Final Week)	Sample Size (n)
DNS Blocking Efficiency (%)	81.2% (3151/3880 queries)	97.0% (weekly aggregate)	Six weekly observations
VPN Connection Time (ms)	~3000 ms (mean)	~2400 ms (mean)	168 handshake measurements
Tunnel RTT (ms)	127.2 ms (mean)	114.8 ms (mean)	126 ICMP samples
Tunnel Uptime (%)	100% (0 unexpected drops)	100%	168 monitoring intervals
Firewall Policy Enforcement	Correct rule enforcement confirmed	Correct rule enforcement confirmed	168 monitoring intervals
Total DNS Queries (n)	210 (Week 1)	1260 (final week)	Six weekly observations
Blocked Domains (n)	275,080 (Week 1)	275,080 (final week)	Six weekly observations

**Table 5:** Statistical summary of network security improvements.

Metric	Baseline (Week 1)	Optimized (Final Week)	95% Confidence Interval [CI]	Statistical Significance (*p*-Value)
DNS Blocking Efficiency (%)	81.2%	97.0% (±1.4%)	[95.6%, 98.4%]	*p* < 0.0001
VPN Latency (Mean RTT)	127.2 ms	114.8 ms (±2.1)	[112.1, 117.5] ms	*p* < 0.001
Connection Time	3012 ms	2410 ms (±45)	[2382, 2438] ms	*p* < 0.01

**Table 6:** Presents a structured comparison between the proposed NetworkGuard architecture and representative IoT security solutions.

Study	Detection Method	Hardware	Integrated VPN	DNS Filtering	Firewall
[32]	Snort IDS	Raspberry Pi 3 B	No	No	Yes
[33]	ML/DL IDS	Raspberry Pi 3 B+	No	No	No
NetworkGuard (This Work)	DNS+ Firewall+ VPN	Raspberry Pi 4 B	Yes	Yes	Yes

## Data Availability

The data presented in this study are available from the corresponding author upon reasonable request due to privacy and security considerations.
